# Variation in Preoperative Refraction Results Among Refractive Surgery Patients: A Comparison of Five Measurement Methods

**DOI:** 10.7759/cureus.108693

**Published:** 2026-05-11

**Authors:** Muhammad Yusuf Abdurrahman, Azuwan Musa, Abd Bari Muhd-Syafi, Khairidzan Mohd. Kamal

**Affiliations:** 1 Department of Ophthalmology, International Islamic University Malaysia, Kuala Lumpur, MYS; 2 Department of Ophthalmology, Kulliyyah of Medicine, International Islamic University Malaysia, Kuantan, MYS

**Keywords:** autorefraction, manifest refraction, refractive surgery, righton retinomax, spherical equivalent, wavefront aberrometry, zeiss i.profiler

## Abstract

Background: Accurate preoperative refraction is critical in refractive surgery planning. Manifest refraction (MR) remains the clinical gold standard but is time-consuming and operator-dependent. Alternative objective methods, including wavefront aberrometry and automated refraction, offer speed and objectivity, yet their agreement with MR varies, particularly regarding astigmatic components. Identifying which alternatives most closely approximate MR has direct clinical implications.

Objective: To compare preoperative spherical equivalent (SE) refraction obtained by five measurement methods and identify which most closely agree with MR as potential clinical alternatives.

Methods: Retrospective comparative study of 104 eyes (52 patients) at IIUM Eye Specialist Clinic (January-May 2021). Five methods were compared: MR, Wavefront Supported Custom Ablation (WASCA) aberrometer, Zeiss i.Profiler (ZIP), Righton Retinomax Handheld (RRH) autorefractor, and focimeter (FOCI). Pairwise comparisons used paired t-tests on SE values.

Results: Significant pairwise differences were found between all five methods (P < 0.05), except ZIP vs. RRH (mean difference (MD) = 0.03 diopters (D); P = 0.753). The two methods with the smallest mean differences from MR were ZIP (MD = 0.20 D) and RRH (MD = 0.22 D), both below the 0.25 D clinical significance threshold. The largest discrepancy was between WASCA and FOCI (MD = 1.44 D).

Conclusion: Both the ZIP and RRH autorefractors produced SE values in close agreement with MR. ZIP is preferred due to superior positional control, yielding more reproducible astigmatic readings. Routine autorefraction with ZIP may supplement MR in preoperative refractive surgery assessment.

## Introduction

Accurate preoperative refraction assessment is an indispensable component of refractive surgery planning. The refractive correction delivered by any laser or surgical procedure is fundamentally predicated on the precision of preoperative measurement, and even small errors in refraction can translate into clinically significant residual refractive error or suboptimal patient satisfaction [1].

Manifest refraction (MR) has long served as the reference standard for determining the refractive error of the eye [2]. It is a subjective technique that integrates objective findings with patient feedback to achieve the best corrected visual acuity. Its reliability is well established: Reinstein et al. demonstrated that interobserver variability in MR performed under a standardised protocol was less than 0.25 diopters (D), confirming its reproducibility across clinicians in a refractive surgery setting [3]. Despite this, MR is time-consuming, operator-dependent, and requires active patient cooperation and communication, characteristics that may introduce variability in certain clinical scenarios [4].

Several objective refraction methods have been developed to supplement or replace MR in selected contexts. Autorefractors, including handheld and tabletop designs, estimate refractive error automatically based on infrared light reflection principles. Multiple studies have reported good agreement between autorefraction and subjective refraction for spherical equivalent (SE), though cylinder measurements frequently show wider limits of agreement [5,6]. Venkataraman et al. demonstrated that autorefractor design features, particularly inbuilt fogging mechanisms, significantly influence both precision and agreement with subjective refraction [7].

Wavefront aberrometry represents a more sophisticated approach, capable of measuring not only lower-order aberrations (sphere and cylinder) but also higher-order aberrations such as coma, trefoil, and spherical aberration [8]. The Wavefront Supported Custom Ablation (WASCA) aberrometer (Carl Zeiss Meditec, Jena, Germany) is one such system, widely used for preoperative planning in laser refractive surgery. Several authors have reported that the WASCA tends to overestimate myopia relative to MR. Reinstein et al. specifically examined the accuracy of WASCA refraction compared to MR in myopic eyes, finding that while concordance was generally high on average, outlier measurements did occur [9]. Cervino et al. further showed that wavefront analysers, including the WASCA-induced instrument myopia, produce readings approximately 0.3-0.5 D more myopic than open-view autorefractors in non-cycloplegic conditions - an effect attributed to accommodation [10]. Huelle et al. confirmed that WASCA measured systematically higher myopic and astigmatic values than MR across a range of patient populations [11].

The ZEISS i.Profiler (ZIP) Plus (Carl Zeiss Vision, Aalen, Germany) is a combined Hartmann-Shack wavefront sensor and corneal topographer that performs objective autorefraction in a controlled, seated position with an internal fixation target. Studies have demonstrated high repeatability for wavefront aberration measurements with this device [12]. Rauscher et al. characterised the repeatability and agreement of the ZIP Plus under cycloplegic and non-cycloplegic conditions, establishing its reliability profile for clinical use [13]. Lebow and Campbell, in the original study that our work cites, directly compared the i.Profiler Plus with a conventional ray-deflection autorefractor (Canon RK-F2) and subjective refraction, finding that both instruments provided clinically useful SE data, although the i.Profiler Plus showed a small systematic bias toward more negative readings [14].

The Righton Retinomax Handheld autorefractor (RRH) (Right Manufacturing, Tokyo, Japan) is a portable device capable of measuring refractive error with flexible patient positioning. Harvey et al. demonstrated that handheld Retinomax autorefraction in children showed repeatability comparable to adult subjective refraction, with mean vector differences of approximately 0.82 D from retinoscopy [15]. The portability of the device introduces operator-dependent variables; however, consistent alignment and a stable measurement axis are harder to maintain compared to fixed tabletop systems.

Despite the existence of individual comparative studies, there is limited evidence directly comparing five concurrent refraction methods within the same preoperative refractive surgery cohort. This study, conducted at IIUM Eye Specialist Clinic, Kuantan, Malaysia, aimed to compare the preoperative SE refraction obtained by MR, WASCA, ZIP, RRH, and focimeter (FOCI), and to determine which alternative method most closely approximates MR as a potential substitute in preoperative refractive surgery assessment.

## Materials and methods

Study design and setting

This was a retrospective comparative study conducted at the IIUM Eye Specialist Clinic, Department of Ophthalmology, Kulliyyah of Medicine, International Islamic University Malaysia. Data were collected from routine preoperative refractive surgery assessments performed between January and May 2021. The study was conducted in accordance with the tenets of the Declaration of Helsinki.

Participants

Adult patients who underwent comprehensive preoperative refractive surgery assessment at the IIUM Eye Specialist Clinic during the study period were eligible. Inclusion criteria required no history of ocular disease or prior corneal surgical procedures. Patients with any ocular comorbidity, incomplete refraction data from any of the five methods, or contact lens wearers who had not observed the required lens cessation period prior to assessment were excluded. A total of 104 eyes from 52 patients met the eligibility criteria and were included in the final analysis.

Refraction methods

Five refraction methods were performed on each participant during the preoperative assessment visit:

Manifest Refraction (MR)

Subjective refraction performed by a trained optometrist or ophthalmologist to determine the best corrected visual acuity using a standard phoropter protocol. This served as the reference standard for all comparisons.

Wavefront Supported Custom Ablation (WASCA) Aberrometer

A Hartmann-Shack wavefront sensor-based system providing objective measurement of refractive error and higher-order aberrations. Measurements were calculated for a 6-mm analysis zone using the Seidel method.

Zeiss i.Profiler (ZIP)

A tabletop combined wavefront aberrometer and corneal topographer with an internal fixation target that provides objective autorefraction in a controlled, seated position.

Righton Retinomax Handheld Autorefractor (RRH)

A portable handheld autorefractor capable of measuring refractive error in a flexible patient position. Measurements were performed by a single trained operator.

Focimeter (FOCI)

Lensometry of the patient's habitual spectacle lenses using a standard FOCI/lensometer to record the current spectacle prescription.

Outcome measure

All refraction results were converted to SE, calculated as: SE = sphere + (cylinder ÷ 2). SE was used as the primary outcome measure for all pairwise comparisons, consistent with established methodology in refraction comparison studies.

Statistical analysis

Pairwise comparisons between all five refraction methods were performed using paired t-tests. A mean difference of less than 0.25 D was used as the pre-specified threshold for clinical equivalence, consistent with previously published interobserver repeatability data for MR. A P-value of less than 0.05 was considered statistically significant. All data are reported as mean (standard deviation, SD). Statistical analyses were performed using IBM SPSS Statistics for Windows, Version 26 (Released 2018; IBM Corp., Armonk, New York, United States).

## Results

Demographic characteristics

A total of 104 eyes from 52 patients were included. The mean age was 30.9 years (SD = 6.89, range: 21-49 years). The majority of participants were female (73.1%, n = 38) and of Malay ethnicity (91.7%, n = 48). All participants had no prior history of ocular disease or corneal surgery.

Pairwise comparison of refraction methods

Statistically significant differences in SE were observed in all pairwise comparisons (P < 0.05) except for the ZIP vs. RRH comparison (MD = 0.03D; P = 0.753). The largest mean difference was between WASCA and FOCI (MD = 1.44D; t = -14.023; P < 0.001), followed by MR and WASCA (MD = 0.82D; t = -8.60; P < 0.001). The smallest clinically meaningful differences from MR were observed for ZIP (MD = 0.20D; P = 0.023) and RRH (MD = 0.22D; P = 0.016), both falling below the 0.25D clinical significance threshold. The complete pairwise comparison data are presented in Table 1.

**Table 1:** Pairwise comparisons were performed using paired t-tests (n = 104 eyes). Data are presented as mean ± SD in diopters (D). * indicates statistical significance at P < 0.05. The total number of eyes is 104. WASCA: Wavefront Supported Custom Ablation; MR: manifest refraction; RRH: Righton Retinomax Handheld autorefractor; ZIP: ZEISS i.Profiler Plus; FOCI: focimeter

Comparison (A vs. B)	Mean ± SD - A (D)	Mean ± SD - B (D)	Mean Difference A-B (D)	t value	P-value
WASCA vs. MR	-5.98 ± 1.87	-5.16 ± 2.08	0.82	-8.600	<0.001*
WASCA vs. RRH	-5.98 ± 1.87	-5.38 ± 2.13	0.60	-6.280	<0.001*
WASCA vs. ZIP	-5.98 ± 1.87	-5.36 ± 2.09	0.62	-5.500	<0.001*
WASCA vs. FOCI	-5.98 ± 1.86	-4.54 ± 1.96	1.44	-14.023	<0.001*
RRH vs. MR	-5.38 ± 2.13	-5.16 ± 2.08	0.22	-2.460	0.016*
RRH vs. ZIP	-5.38 ± 2.13	-5.36 ± 2.09	0.02	-0.315	0.753
RRH vs. FOCI	-5.38 ± 2.13	-4.54 ± 1.96	0.84	-8.210	<0.001*
ZIP vs. MR	-5.36 ± 2.09	-5.16 ± 2.08	0.20	-2.305	0.023*
ZIP vs. FOCI	-5.36 ± 2.09	-4.54 ± 1.96	0.82	-8.480	<0.001*
FOCI vs. MR	-4.54 ± 1.96	-5.16 ± 2.08	0.62	9.780	<0.001*

## Discussion

This retrospective comparative study examined the agreement between five preoperative refraction methods in a cohort of refractive surgery candidates at a Malaysian academic ophthalmology centre. The principal finding was that both ZIP and RRH demonstrated SE values closest to MR, with mean differences of 0.20 D and 0.22 D, respectively, both falling below the 0.25 D threshold for clinical significance established in interobserver MR reproducibility studies by Reinstein et al. [3]. These results suggest that both instruments are viable clinical alternatives to MR for SE estimation in the preoperative setting.

The WASCA aberrometer demonstrated the greatest divergence from MR (MD = 0.82 D). This finding is consistent with previously published evidence. Reinstein et al. evaluated WASCA accuracy in a myopic cohort undergoing refractive surgery and found that while average concordance was high, outlier measurements occurred [9]. Cervino et al. demonstrated that wavefront analysers, including the WASCA, systematically induced instrument myopia, producing SE readings 0.3 to 0.5 D more negative than open-view autorefractors due to residual accommodation in non-cycloplegic conditions [10]. In our cohort, the WASCA mean SE of -5.98 D compared to the MR mean of -5.16 D (MD = 0.82 D) closely aligns with these published findings. Huelle et al. similarly confirmed that WASCA measured systematically higher myopic values than MR, especially in higher myopes [11]. Importantly, Lebow and Campbell, in the study originally cited in our poster, noted that the ZIP Plus provided slightly more negative readings than subjective refraction (MD = 0.11 D in their cohort vs. 0.20 D in ours), suggesting the more-minus bias is a class effect seen across wavefront-based instruments, though of smaller magnitude with the i.Profiler compared to the dedicated WASCA aberrometer [14].

The absence of a statistically significant difference between ZIP and RRH (MD = 0.03 D; P = 0.753) indicates strong interchangeability between these two instruments for SE measurement. This finding is clinically meaningful, as it demonstrates that the two most MR-concordant instruments produce equivalent outcomes, offering flexibility in clinical workflow. Kemchoknatee et al. similarly found that multiple autorefractors can produce comparable SE values, with the primary limitation lying in cylinder measurement reproducibility [5]. Paudel et al. confirmed that the Nidek ARK-1 autorefractor provides a reasonable and repeatable estimate of refractive error for SE, although their study likewise highlighted that cylinder values may exhibit wider limits of agreement [6].

The systematic over-estimation of myopia by the WASCA has important clinical implications. When wavefront aberrometry is used to directly program a laser ablation, as is the case with wavefront-guided treatments, any systematic myopic bias in the aberrometric refraction will translate directly into under-correction. This concern was specifically quantified by Reinstein et al., who modelled predicted LASIK outcomes using WASCA aberrometric versus MR in 869 myopic and 413 hyperopic eyes, finding that MR produced better refractive accuracy, with 81% of myopic eyes within ±0.50 D of target using MR versus only 67-70% using WASCA-derived refraction [2]. Saad and Frings, in a more recent multicentre study comparing WASCA and Sirius wavefront aberrometry against subjective refraction in PRK patients, further confirmed pre-operative underestimation of myopia by wavefront systems [16].

The FOCI showed the second largest divergence from MR (MD = 0.62 D, FOCI vs. MR). This was expected, as FOCI measures the power of the patient's existing spectacle lenses rather than the current refractive state of the eye. Spectacle prescriptions may be outdated, undercorrected, or may not reflect interval refractive changes, particularly in younger myopes whose refraction may progress significantly between spectacle dispensing episodes. For this reason, focimetry should not be used as a primary preoperative refraction method in the context of refractive surgery.

The practical distinction between ZIP and RRH, however, lies in the measurement of astigmatism rather than SE. The ZIP operates in a fixed, tabletop configuration with a controlled internal fixation target. Its positional stability eliminates the operator-dependent variation in head tilt and instrument angle that is inherent to handheld devices. Venkataraman et al. demonstrated that inbuilt fogging, a feature present in the ZIP, is the most important design characteristic for improving both precision and accuracy of objective refraction, particularly for astigmatic components [7]. In contrast, the Retinomax, while highly portable and suitable for special populations such as young children or non-cooperative patients, as demonstrated by Harvey et al. [15], requires an experienced and steady operator to minimise measurement variability of the astigmatic axis and cylinder power. Liao et al. confirmed that the i.Profiler Plus demonstrates high repeatability for wavefront aberration measurements, including higher-order aberrations in healthy eyes [12]. For these reasons, while both ZIP and RRH are acceptable alternatives for SE estimation, ZIP is preferred in a preoperative refractive surgery setting where accurate cylinder determination is of equal or greater importance to sphere measurement.

The demographic composition of our sample - predominantly young, female, and Malay patients - reflects the typical patient population seeking refractive surgery at IIUM Eye Specialist Clinic. Comparably young mean ages (30.9 ± 6.89 years) are consistent with published refractive surgery cohorts from Southeast Asian centres. The high female predominance may partly reflect healthcare-seeking behaviour patterns in the local context. Ethnic homogeneity limits the generalisability of the findings to more diverse populations.

Several limitations should be acknowledged. The retrospective design introduces potential selection bias, and the absence of cycloplegic refraction as a comparator limits interpretation, as non-cycloplegic MR may itself be subject to accommodative variation, particularly in the younger age groups represented in this cohort. The study did not assess repeatability or intra-session variability for individual instruments, and Bland-Altman analysis for limits of agreement - the recommended method for method comparison studies - was not performed. Future prospective studies with Bland-Altman analyses, repeatability assessments, and more diverse samples would strengthen the evidence base. Additionally, inclusion of cylinder and axis analysis would provide more complete clinical guidance.

## Conclusions

Both the ZIP and the RRH demonstrated SE measurements in close clinical agreement with MR, with mean differences below the 0.25 D threshold for clinical significance. Neither the WASCA aberrometer nor FOCI should be used as standalone alternatives to MR for preoperative refractive surgery assessment, given their substantially larger divergences. ZIP is recommended as the preferred alternative due to its fixed-position measurement system, internal fixation target, and superior repeatability for astigmatic components. Incorporation of ZIP alongside MR in a standard preoperative assessment protocol may improve workflow efficiency without compromising measurement accuracy.
